# Neuronal Nitric Oxide Synthase Induction in the Antitumorigenic and Neurotoxic Effects of 2-Methoxyestradiol

**DOI:** 10.3390/molecules190913267

**Published:** 2014-08-28

**Authors:** Magdalena Gorska, Alicja Kuban-Jankowska, Michal Aleksander Zmijewski, Monika Gorzynik, Michal Szkatula, Michal Wozniak

**Affiliations:** 1 Department of Medical Chemistry, Medical University of Gdansk, Gdansk 80-211, Poland; E-Mails: alakuban@interia.pl (A.K.-J.); monika.gorzynik@urz.pl (M.G.); mszkatula@gumed.edu.pl (M.S.); mwozniak@gumed.edu.pl (M.W.); 2 Department of Histology, Medical University of Gdansk, Gdansk 80-211, Poland; E-Mail: mzmijewski@gumed.edu.pl

**Keywords:** osteosarcoma, hippocampus, cancer, neurodegenerative disease, 2-methoxyestradiol, neuronal nitric oxide synthase, 3-nitrotyrosine

## Abstract

*Objective*: 2-Methoxyestradiol, one of the natural 17β-estradiol derivatives, is a novel, potent anticancer agent currently being evaluated in advanced phases of clinical trials. The main goal of the study was to investigate the anticancer activity of 2-methoxy-estradiol towards osteosarcoma cells and its possible neurodegenerative effects. We used an experimental model of neurotoxicity and anticancer activity of the physiological agent, 2-methoxyestradiol. Thus, we used highly metastatic osteosarcoma 143B and mouse immortalized hippocampal HT22 cell lines. The cells were treated with pharmacological (1 μM, 10 μM) concentrations of 2-methoxyestradiol. *Experimental*: Neuronal nitric oxide synthase and 3-nitrotyrosine protein levels were determined by western blotting. Cell viability and induction of cell death were measured by MTT and PI/Annexin V staining and a DNA fragmentation ELISA kit, respectively. Intracellular levels of nitric oxide were determined by flow cytometry. *Results*: Here we demonstrated that the signaling pathways of neurodegenerative diseases and cancer may overlap. We presented evidence that 2-methoxyestradiol, in contrast to 17β-estradiol, specifically affects neuronal nitric oxide synthase and augments 3-nitrotyrosine level leading to osteosarcoma and immortalized hippocampal cell death. *Conclusions*: We report the dual facets of 2-methoxyestradiol, that causes cancer cell death, but on the other hand may play a key role as a neurotoxin.

## 1. Introduction

Osteosarcoma (OS) is one of the most malignant bone tumors of childhood and adolescence. Despite remarkable improvements in OS therapy implemented in the last few decades, there has been no significant progress since 1970 when series of clinical trials were launched at the Memorial Sloan-Kettering Hospital and neoadjuvant therapy was introduced to treatment. Then the 5-year survival rate reached approx. 70% [1,2,3,4,5]. The difficulties in effective treatment of OS are associated with its metastatic potential and chemoresistance, thus the searchfor novel, potent anticancer drugs is a scientific task of the highest priority. A great number of studies indicate that carcinogenesis and angiogenesis can be influenced by 17β-estradiol metabolites [6,7]. 2-Methoxyestradiol (2-ME), one of the natural 17β-estradiol derivatives, is a novel potentially active anticancer agent [8,9,10,11]. 2-ME is active after oral administration and in pharmacological, nontoxic doses affects cancer cell growth both *in vivo* and *in vitro*, as well as leading to inhibition of metastatic processes (breast cancer, pancreas cancer, Ewing sarcoma, osteosarcoma) [12,13,14,15]. 2-ME (branded as Panzem) is currently being evaluated in ongoing advanced phases of clinical trials in patients with breast cancer, ovarian cancer and prostate cancer or multiple myeloma [8,9,10,11,12,13,14,15,16,17,18,19]. 2-ME is well tolerated by patients. The adverse reactions of 2-ME are mild and usually involve nausea, vomiting, diarrhea, flushing and headaches [8,9,10,11,16,17,18,19]. According to Sutherland *et al.* 2-ME inhibits cell proliferation at threshold concentrations of 0.1–0.3 μM, reaching its maximum effect at 10–20 μM [20]. Interestingly, normal cells are more resistant to the proapoptotic properties of 2-ME [21,22]. Thus, 2-ME was demonstrated as a relatively safe anticancer agent. Nonetheless, some adverse drug reactions like neurotoxic effects are observed only after long-term administration. Interestingly, a recent study suggested that the molecular pathways of neurodegeneration and cancer may overlap [23]. Several neurodegeneration-causing factors like PARK2 parkin, and PARK5 have been recently been evidenced to be involved in cancer development by playing an important role as regulators of cell cycle [23,24]. In the same context, α- and β-synucleins may play an important role in cancer pathogenesis, regulation and tumor differentiation, including osteosarcoma [25,26]. Furthermore, it was also demonstrated that selectively nitrated α-synuclein is directly associated with nitro-oxidative stress-induced damage and progression of neurodegenerative diseases [26]. Reactive nitrogen species (RNS) play an important role in mediating cell signaling pathways, but their increased concentration leads to nitro-oxidative stress resulting in cell cycle arrest or cell death. Nitric oxide is synthetized by the action of nitric oxide synthases (NOSs), a group of hemoproteins that catalyze oxidation of l-arginine to citrulline releasing a molecule of nitric(II) oxide (NO) [27]. Three isoforms of NOS can be distinguished: nNOS (neuronal nitric oxide synthase) found mainly in neurons, iNOS (inducible nitric oxide synthase) induced by many stimuli like stress or inflammation in different kinds of cells and tissues, and eNOS (endothelial nitric oxide synthase) expressed mainly in endothelial cells [27]. Although nitric oxide is not highly reactive, it may easily react with oxygen radicals to generate highly damaging RNS like peroxynitrite or nitrogen dioxide. 3-Nitrotyrosine (3-NT) formed in the reaction of nitrating oxidants with protein tyrosine residues or free tyrosine is a fingerprint of RNS [28]. Due to that fact 3-NT is an indicator of nitro-oxidative stress under pathophysiological conditions. Interestingly, increased levels of nitrated proteins and 3-NT have been associated with a variety of neurodegenerative diseases like Parkinson’s disease (PD) [29]. Although 3-NT is considered as a biological marker of RNS, it has been also recently referred to as a neurotoxin [30,31]. However, the molecular mechanism of 3-NT-induced apoptosis still needs to be elucidated.

Due to the fact that glutamergic system and NMDA receptor subunits (NR1, NR2A, NR2B, NR2D) were identified in OS cells [32,33], the main objective of the present study was to determine a plausible link between the molecular mechanisms of neurodegenerative diseases and cancer. For this purpose we used highly metastatic human osteosarcoma 143B cell lines as a model of cancer. Moreover, a mouse immortalized hippocampal HT22 cell line was employed in order to evaluate the neurotoxicity of the compound [34,35,36,37]. Herein, we evidenced that 2-methoxyestradiol (2-ME), a physiological derivative of 17β-estradiol (E2), may induce OS 143B and neuronal HT22 cell death via induction of nitro-oxidative stress.

## 2. Results and Discussion

The interesting notion of the existence of an overlap between neurodegenerative diseases and cancer has been recently demonstrated [23]. Furthermore, it has been suggested that neurodegenerative diseases, mainly Parkinson’s disease, and tumors are linked by a negative interaction [23]. Interestingly, this connection concerns not only CNS tumors, but also peripheral ones.

### 2.1. Anticancer Effects of 2-ME

#### 2.1.1. Antiproliferative Properties of 2-ME

**Figure 1:**
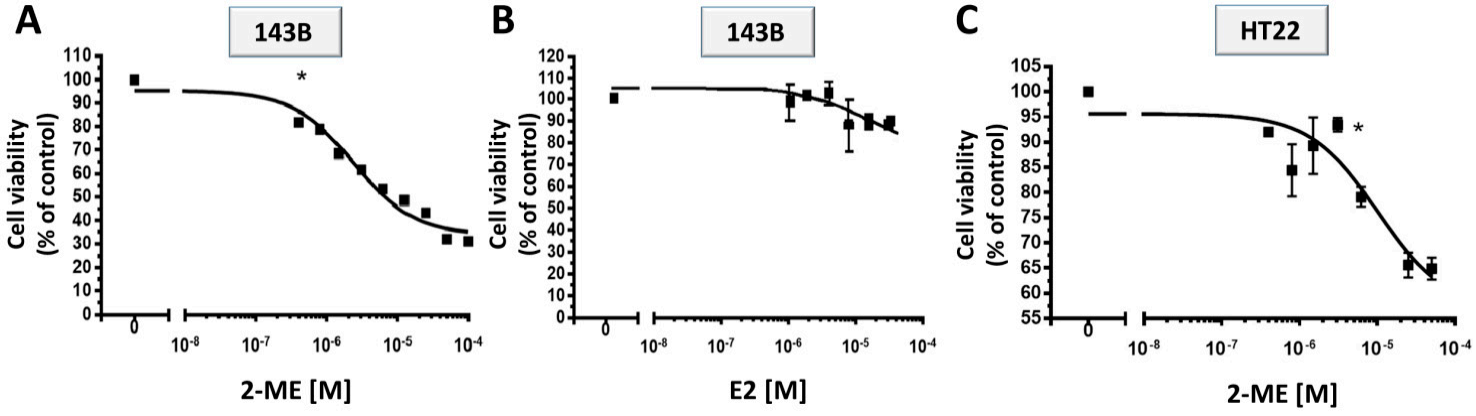
Impact of treatment with 2-ME and E2 on OS cell death. (**A**,**B**) Inhibition of cell viability by 2-ME and E2; (**C**) Inhibition of cell viability HT22 cells by 2-ME. OS 143B and immortalized hippocampal HT22 cells were treated with a series of dilutions (0.8–50 μM) of 2-ME or E2. The cell proliferation was determined by MTT assay. 2-ME inhibited 143B cell proliferation in contrast to E2. Data are presented as the mean ± SE values form at least three independent experiments. Data were analyzed performing One-way ANOVA combined with Dunnett’s Multiple Comparison Test; * *p* < 0.01 *versus* control (c).

The original viewpoint on E2 metabolites as being biologically inactive excretion products has been rejected by numerous research findings [7,13,14]. Preclinical research suggests a wide spectrum of possible anticancer mechanisms of action of 2-ME that seem to be directly associated with the inhibition of angiogenesis and induction of apoptosis in tumorous and proliferating cells [7,13,14]. The first goal of the study was to determine the antiproliferative effects of 2-ME treatment towards cancer OS and immortalized hippocampal cells. 143B and HT22 cells were treated with a series of dilutions (0.8–50 μM) of 2-ME for 24 h. Subsequently, the inhibition of cell growth was observed by means of an MTT-assay. As presented in Figure 1, 2-ME effectively inhibited OS and hippocampal cell growth in a concentration-dependent manner. Viability of 143B cells was significantly diminished from 81% to 31% in the presence of 2-ME (0.8–50 μM) as compared to control (c) (Figure 1A). As presented in Figure 1B, E2 did not exert any statistically significant antiproliferative effect on OS cells. Proliferation of immortalized hippocampal HT22 cells was inhibited from 92% to 60% in the presence of 2-ME (0.8–50 μM) as compared to control (c) (Figure 1C).

#### 2.1.2. Effect of 2-ME and E2 on Induction of Cell Death in OS 143B

**Figure 2:**
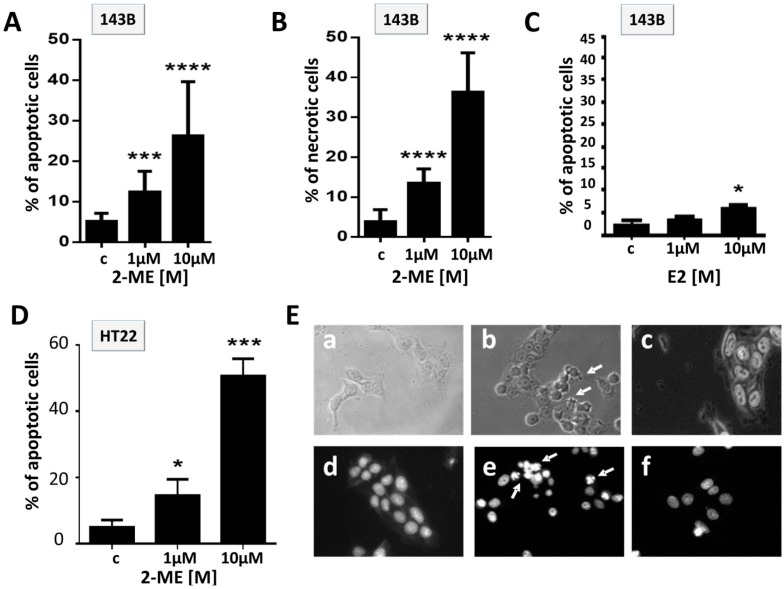
Impact of treatment with 2-ME on hippocampal cell death. (**A**–**C**) Induction of cell death by 2-ME and E2 in OS cells. 143B osteosarcoma cells were treated with vehiculum (c), 2-ME (1 μM, 10 μM) or E2 (1 μM, 10 μM) for 24 h; (**D**) Induction of cell death by 2-ME in immortalized hippocampal cells. HT22 cells were treated with solvent (c), 2-ME (1 μM, 10 μM) for 24 h. The cells were harvested and the percentage of apoptotic and necrotic cells was determined by PI-Annexin staining. Values are the mean ± SE of three independent experiments (N = 6 replicate cultures). Data were analyzed performing Student’s *t* tests. * *p* < 0.1, ** *p* < 0.01, *** *p* < 0.001, **** *p* < 0.0001 *versus* control (c); (**E**) Morphological changes (**a**–**c**) and Hoechst 33.258 staining (**d**–**f**) observed in control (c); (a), (d), 1 μM 2-ME; (b), (e), 1 μM E2; (c), (f), 8 h treated cells. A representative experiment out of three performed is shown.

The next goal of the study was to determine the induction of cell death in OS and HT22 cells by 2-ME. 143B cells were treated with 2-ME or E2 at concentrations of 1 μM and 10 μM for 8 h and 24 h (Figure 2A–C) to determine the proapoptotic properties of 2-ME. Percent of apoptotic cells after 24 h incubation with 1 μM 2-ME was statistically elevated up to 10%, while it dramatically increased to 36.3% ± 3.5% after 24 h treatment with 10 μM 2-ME (Figure 2A). The level of necrotic cells detected after 24 h incubation of OS cells with 1 μM, 10 μM 2-ME was equal to 12.3% ± 1.5% and 26.2% ± 4%, respectively (Figure 2B). E2 induced apoptosis (6% of apoptotic cells) only when used at the concentration of 10 μM, whereas 1 μM E2 did not affect the induction of OS cell death (Figure 2C). Morphological changes characteristic of apoptosis (from irregular shape into a rounded shape) were observed after 8 h of treatment with 2-ME, while after 8 h of incubation with E2 we did not notice perturbed cell morphology (Figure 2E). Additionally, we observed a massive induction of cell death after 24 h treatment of immortalized hippocampal HT22 cells with 2-ME (1 μM, 10 μM, Figure 2D). The obtained data suggest that 2-ME exerts both anticancer and neurotoxic effects at pharmacological concentrations. Though, normal cells are thought to be more resistant to proapoptotic properties of 2-ME [21,22], we demonstrated that toxic effect of 2-ME may be dependent on cellular context. Our results support data obtained by the Picazo research group that indicated the neurotoxicity of the agent [38]. Though, 2-ME is well tolerated by patients and the adverse reactions of 2-ME are mild and usually involve nausea, vomiting, diarrhea, flashing, headache [8,9,10,11,16,17,18,19], we suggest possible long-term side effects of the agent such as memory disorders or depression.

### 2.2. Neuronal-Type Signaling in OS Cells—nNOS as Molecular Target of 2-ME

#### 2.2.1. Impact of 2-ME on nNOS and 3-NT Protein Levels

**Figure 3:**
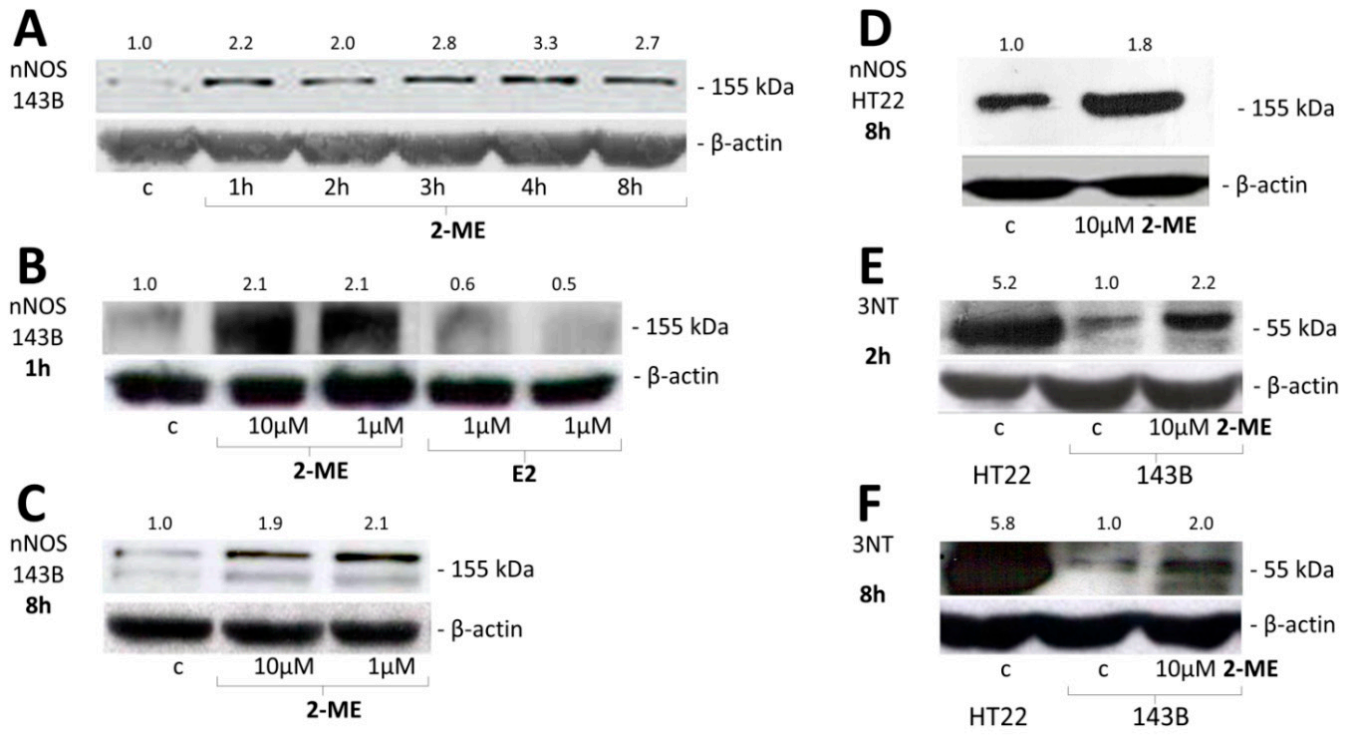
2-ME increased nNOS and 3-NT protein level in OS 143B and hippocampal HT22 cells. (**A**) Time-dependent induction of protein level of nNOS by treatment of OS 143B cells with 1 μM 2-ME; (**B**) 2-ME (1 μM, 10 μM) upregulated nNOS protein after 1 h incubation of OS 143B cells. Treatment of OS 143B cells with E2 (1 μM, 10 μM) for 1 h decreased the enzyme protein level; (**C**,**D**) 2-ME (1 μM, 10 μM) upregulated nNOS protein after 8 h of incubation of OS 143B and immortalized hippocampal HT22 cells; (**E**,**F**) 2-ME (1 μM, 10 μM) increased level of 3-NT in OS cells. HT22 cells are characterized by constitutive high level of 3-NT.

**Figure 4:**
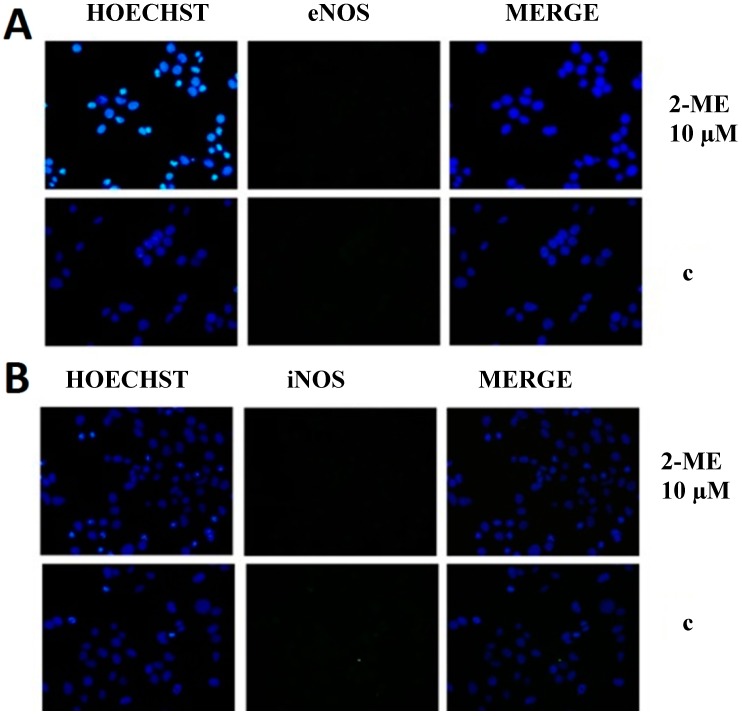
2-ME does not affect eNOS (**A**) and iNOS (**B**) protein level in OS 143B cells. 143B OS cells were treated with vehicle (c) or 2-ME (10 μM) for 8 h; The levels of eNOS (**A**), iNOS (**B**) protein were determined by immunofluorescence. Cell nuclei are shown in blue while NOSs immunoreactivity is seen in green. Merged images of both were also presented. Each experiment was performed at least three times. The representative data are shown. Original magnification is 40.

Next, we revealed that molecular target for 2-ME in OS and immortalized hippocampal cells is a neuronal isoform of nitric oxide synthase (nNOS). *In vivo* studies revealed glutamate receptor activity in tissues such as bone, and NMDA receptor subunits were identified in OS MG63 cells [32,33]. Herein, we have evidenced, to our knowledge for the first time, neuronal-like signaling in non-neuronal highly metastatic human OS 143B cells. Moreover, while nNOS has been believed so far to be only a constitutive isoform of the NOS enzyme, we are presenting evidence of its inducible character [39]. 2-ME used at concentration of 1 μM selectively increased nNOS protein levels in a time-dependent manner. The level of enzyme was elevated after 1 h incubation with 1 μM 2-ME (2.2-fold as compared to control (c)) with a maximal 3.3-fold increased peak after 4 h what was maintained till 8 h of incubation with stimulus (Figure 3A–C). Additionally, E2 lowered the protein level of nNOS in OS cells as compared to 2-ME (Figure 3B). Moreover, the induction of neuronal isoform of NOS by 2-ME seems to be isoform- specific, as 2-ME did not affect eNOS and iNOS protein levels (Figure 4).

Interestingly, the mode of action of 2-ME is specific not only for cancer OS cells, but also hippocampal cells. 2-ME also increased nNOS protein levels in HT22 cells characterized by constitutive enzyme expression (Figure 3D). The obtained data suggest that nNOS should be also considered as inducible isoform of NOS regulated by 2-ME. In fact, Tsukamoto and co-workers reported that 2-ME increased NOS and linked the production of NO with enhanced apoptosis of endothelial cells [40]. Interestingly enough, levels of nNOS were also elevated by 2-ME in HT22 cells what was correlated with enhanced neuronal cell death after 24 h treatment with 2-ME (Figure 2). The neurotoxic effects of 2-ME have been previously demonstrated by Picazo *et al.* and may explain plausible side effects like memory disorders of 2-ME treated patients. The Picazo research group noticed that a number of neurons in the hilus of the dentate gyrus of ovariectomized rats were killed by 2-ME [38].

2.2.2. nNOS Is Engaged in 2-ME Induced Cell Death

**Figure 5:**
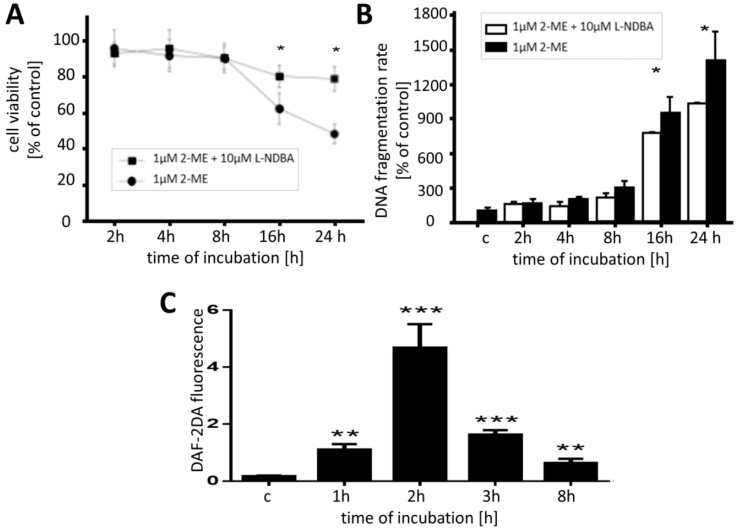
Significance of nNOS in 2-ME-induced apoptosis. (**A**) Cell viability assay (MTT) revealing the statistically significant influence of pretreatment with 10 μM l-NDBA for 1 h on inhibition of OS 143B cell growth after 16 h of 1 μM 2-ME stimulation. The data are presented as a percentage of control (c); (**B**) Pretreatment with 10 μM l-NDBA for 1 h significantly decreased the accumulation of oligonucleosomes in the cellular lysates of OS 143B treated with 2-ME. Statistically significant differences were noted after 16 h of incubation with 2-ME. The results are given as percentage of the control (c); (**C**) Level of intracellular nitric oxide in 143B cells incubated with 1 μM 2-ME. 143B cells were incubated in a time dependent manner with 1 μM 2-ME. Afterwards, intracellular level of nitric oxide was determined with usage of specific detector 4,5-diaminofluorescein diacetate (DAF-2DA) by means of flow cytometry. 2-ME statistically significant increased level of nitric oxide in a time dependent manner with maximal peak at 2 h of incubation.

In order to confirm that nNOS participates in the cell killing mechanism of 2-ME in the OS cell death model used in our studies we used *N*^ω^-Nitroarginine-2,4-l-diaminobutyric amide di(trifluoroacetate) salt (l-NDBA) that selectively blocks NO production in the cells by inhibition of nNOS. OS 143B cells were pretreated for 1 h with 10 μM l-NDBA and subsequently, treated with 1 μM 2-ME in a time-dependent manner. Cell viability was then determined by means of a MTT assay. Pretreatment of 143B cells with l-NDBA significantly increased the mean survival of the cells in 2-ME-treated cultures by 18% or 31%, after 16 h or 24 h of incubation, respectively (Figure 5A). Next, an enzyme-linked immunosorbent assay (ELISA) was performed for the quantitative determination of cytoplasmic histone-DNA fragments. As shown in Figure 5B, pretreatment of the OS cells with the nNOS inhibitor significantly decreased 2-ME-induced apoptosis. The extent of apoptosis observed in the cell cultures 1 h pretreated with 10 μM l-NDBA decreased by approximately 25% after 16 h and approx. 36% after 24 h, as compared to the cells treated with 2-ME alone (Figure 5B). In our studies, both the MTT viability assay and the assessment of DNA fragmentation, revealed that pretreatment with l-NDBA at the concentration of 10 μM for 1 h only partially protected the cells against 2-ME-induced cytotoxicity. These data strongly suggest the vital involvement of nNOS in the 2-ME proapoptotic signalling pathway.

Data were analyzed performing the Student’s *t*-test, if allowed by distribution, and presented as mean values ± SD from three independent experiments, each performed in triplicate. * *p* < 0.05, ** *p* < 0.01, *** *p* < 0.001 *versus* control (c).

### 2.3. RNS Engaged in Anticancer Mechanism of 2-ME

#### Impact on Level of Nitric Oxide

Enhanced activity of nNOS induced by 2-ME in 143B cells was confirmed by means of flow cytometry and a specific fluorescent detector–DAF-2DA–by the augmented levels of nitric oxide after incubation with stimuli. Generation of nitric oxide in 143B OS cells appeared to be dependent on time of incubation. Intracellular nitric oxide was increased 6-fold after 1 h incubation with1 μM 2-ME as compared to control (c) (1 h; 1.1 ± 0.2 *versus* control; 0.17 ± 0.03) (Figure 5C). The highest level of nitric oxide formation was observed after 2 h incubation with 1 μM 2-ME (2 h; 4.68 ± 0.83 *versus* control; 0.17 ± 0.03). After 2 h of incubation the level of intracellular nitric oxide was dramatically decreased. Nonetheless at each time point beyond 4 h a significant generation of nitric oxide after treatment with 1 μM 2-ME was detected as compared to control (c) (Figure 5C). 2-ME-induced DNA-damage (Figure 4B) seems to be a result of nitric oxide generation in OS cells. The association between upregulation of nitric oxide synthase in neurodegeneration disease has been previously described [41,42,43,44,45,46,47]. It has been recently reported that apoptosis of cerebellar granule neurons is triggered by the lowering of extracellular potassium levels and is dependent on both nNOS induction and a subsequent nitric oxide overproduction [47]. Moreover, the possible link between CB(2) receptors, nitric oxide synthases, nitro-oxidative stress, and cell death during neurodegeneration was reported by Pacher and Mackie [45]. While exogenous nitric oxide can induce apoptosis, it remains unclear if endogenously produced nitric oxide plays an important signaling role in cells undergoing apoptosis in response to various physiological stimuli [47]. Adjuvant role of RNS in anticancer therapy due to cancer cells chemosensitization has been widely reported [48]. One of the mechanisms of the pathogenic role of nitric oxide is its reaction with superoxide anion leading to peroxynitrite and consequently, nitrogen dioxide formation. Superoxide dismutase (SOD) competes with nitric oxide prevents generation of derivatives of nitric oxide (peroxynitrite, nitrogen dioxide) that may modify proteins to form 3-NT. Indeed, free 3-NT and similarly modified proteins are footprints of nitro-oxidative stress under pathophysiological conditions. It has been also revealed that nNOS plays an important role in nitration and thus inactivation of SOD in CNS [49].

Here, we demonstrated the common feature of neuronal and 2-ME-treated OS cells that is elevated level of 3-NT. The obtained data of increased nNOS induced by 2-ME in OS cells are strictly correlated to elevated 3-NT expression. The level of 3-NT was enhanced by approx. 2-fold as soon as after 2 h and maintained till 8 h of incubation with stimuli (Figure 3E,F). As demonstrated in Figure 2E,F HT22 are characterized by high level of 3-NT (approx. 5-fold higher as compared to OS 143B cells) plausibly due to constitutive nNOS expression. We observed nitric oxide production followed by cell death of 2-ME treated OS cells possibly associated with nitric oxide-DNA damage and elevated level of 3-NT. One of the proteins that may play an important role in neurodegenerative diseases development and tumor growth regulation is α-synuclein, additionally involved in OS differentiation [25]. Nitrated α-synuclein was reported to form brain lesions, a hallmark of neurodegenerative disorders, and the impact of nitrated α-synuclein in OS cells in currently under our investigation. Nonetheless, Fuhjita and co-workers revealed its inhibitory influence on proteasome activity, alterations in protein kinase C signaling pathway and an autophagy lysosomal degradation system resulting in OS MG63 differentiation [23]. Thus, it suggests that tumor growth and differentiation may overlap with neurological system.

## 3. Experimental Section

### 3.1. Reagents

Tissue culture media, antibiotic cocktail, foetal bovine serum, 2-methoxyestradiol, the nNOS inhibitor *N*^ω^-Nitroarginine-2,4-l-diaminobutyric amide di(trifluoroacetate) salt (l-NDBA) were purchased from Sigma-Aldrich (Poznan, Poland). Diaminofluorescein-FM diacetate (DAF-FM DA) and peroxidase-labeled rabbit anti-mouse IgG antibodies were obtained from Molecular Probes (Warsaw, Poland). Monoclonal antibodies against nNOS isoforms were obtained from BD Biosciences (Warsaw, Poland). Horseradish peroxidase-conjugated antibodies against beta-actin were purchased from Santa Cruz Biotechnology (Heidelberg, Germany).

### 3.2. Cell Line and Culture Conditions

The human highly metastatic OS 143B cell line (ATTC-8303) was obtained from the American Tissue Type Collection (Lomianki, Poland). Immortalized mouse hippocampal HT22 cell line was kindly gifted by Tilman Grune from Institute of Nutrition, Department of Nutritional Toxicology, Friedrich Schiller University (Jena, Germany) and by Kelvin Davies from University of Southern California (USC, Los Angeles, CA, USA). The cells were cultured at 37 °C in a humidified atmosphere saturated with 5% CO_2_ using Dulbecco’s Modified Eagle’s medium supplemented with 10% heat-inactivated fetal bovine serum and a penicillin (100 mg/mL)/streptomycin (100 mg/mL) cocktail (Sigma-Aldrich).

### 3.3. Cell Viability Assay (MTT Assay)

OS 143B cells were treated with serial 2-ME dilutions (within the range of 0.8–50 μM) of 2-ME for 24 h. The MTT assay was performed as previously described [50]. The results were presented as a percentage of control (c). Each experiment was performed at least three times.

### 3.4. Assessment of Apoptosis by Flow Cytometry with Double Annexin V—Propidium Iodide (PI) Staining

OS 143B cells were seeded onto six-well plates at a density of three hundred thousand cells/well. After 24 h of culturing in the standard medium, the cells were treated with 2-ME for 24 h. The annexin V- Pi staining was performed as previously described [50]. The results were then analyzed by the Cyflogic software, version 1.2.1. Each experiment was performed at least three times.

### 3.5. Assessment of Apoptosis by DNA Fragmentation ELISA Kit

Cell lysates were prepared from OS 143B cells treated with of 1 μM 2-ME or pretreated for 2 h with 10 μM l-NDBA and treated with 1 μM 2-ME for 2 h up to 24 h. Analysis of apoptosis was performed using a Cell Death Detection ELISA^PLUS^ kit (Roche, (San Francisco, CA, USA)) according to the manufacturer’s protocol.

### 3.6. Western Blotting

Equal amounts of total cell lysates were resolved by 7% (nNOS) and 10% (3-NT) SDS-PAGE. The membranes were then incubated with primary antibodies anti-nNOS (BD Biosciences) (1:1000), anti-3-NT (Sigma-Aldrich) (1:10,000) overnight at 4 °C and next, analysis performed as previously described [50]. The protein level was quantified by densitometry technique using the Quantity one 4.5.2 software. The protein levels, as determined by chemiluminescent quantification, were normalized relative to beta-actin levels found in the samples. Each experiment was performed at least three times.

### 3.7. Immunofluorescence Microscopy

The immunofluorescense was performed as previously described [50]. The cells were treated with 2-ME for 8 h. Anti-eNOS, anti-iNOS (1:50 in 0.3% GSA, 2 h incubation, BD Biosciences) and goat anti mouse secondary-conjugated with CY3 (1:100, GAM Cy3, 1 h incubation, Jackson Immunoresearch, Suffolk, UK) antibodies were used. The images were analysed and merged employing the ImageJ software 1.44p. Each experiment was performed at least three times.

### 3.8. Statistical Analysis

Data are presented as the mean ± SE values form at least three independent experiments. Data were analysed using GraphPad Prism (GraphPad Software, Inc., version 6.03, La Jolla, CA, USA). Significant differences between groups were determined by One-way ANOVA combined with Dunett’s Multiple Comparison test or Student’s *t*-test.

## 4. Conclusions

Though the mechanisms of 2-ME action is still undefined, the drug is currently under clinical trials as a chemotherapeutic agent [8,9,10,11]. Herein, we presented data suggesting that 2-ME when used at pharmacological concentrations acts as effective anticancer agent, but also as plausible neurotoxin towards hippocampal HT22 cells. Thus, we hypothesize plausible side effects of chemotherapy with 2-ME like memory disorders or depression associated with loss of hippocampal cells.

## Abbreviation

c: Control
CNS: Central nervous system
DAF-2DA: Diaminofluorescein-2-diacetate
E2: 17β-Estradiol
l-NDBA: *N*^ω^-Nitroarginine-2,4-l-diaminobutyric amide di(trifluoroacetate) salt
2-ME: 2-Methoxyestradiol
nNOS: Neuronal nitric oxide synthase
3-NT: 3-Nitrotyrosine
NMDA: N-Methyl-d-aspartate receptor
OS: Osteosarcoma
PD: Parkinson’s disease
SOD: Superoxide dismutase
